# Discrimination and Other Barriers to Accessing Health Care: Perspectives of Patients with Mild and Moderate Intellectual Disability and Their Carers

**DOI:** 10.1371/journal.pone.0070855

**Published:** 2013-08-12

**Authors:** Afia Ali, Katrina Scior, Victoria Ratti, Andre Strydom, Michael King, Angela Hassiotis

**Affiliations:** 1 Mental Health Sciences Unit, University College London, London, United Kingdom; 2 Division of Psychology and Language Sciences, University College London, London, United Kingdom; Nathan Kline Institute and New York University School of Medicine, United States of America

## Abstract

**Background:**

People with intellectual disability have a higher prevalence of physical health problems but often experience disparities in accessing health care. In England, a number of legislative changes, policies and recommendations have been introduced to improve health care access for this population. The aim of this qualitative study was to examine the extent to which patients with intellectual disability and their carers experience discrimination or other barriers in accessing health services, and whether health care experiences have improved over the last decade years.

**Method and Main Findings:**

Twenty nine participants (14 patient and carer dyads, and one carer) took part in semi-structured interviews. The interviews were audio-taped and transcribed and analysed using thematic analysis. Eight themes were identified. Half the participants thought that the patient had been treated unfairly or had been discriminated against by health services. There were accounts of negative staff attitudes and behaviour, and failure of services to make reasonable adjustments. Other barriers included problems with communication, and accessing services because of lack of knowledge of local services and service eligibility issues; lack of support and involvement of carers; and language problems in participants from minority ethnic groups. Most participants were able to report at least one example of good practice in health care provision. Suggestions for improving services are presented.

**Conclusion:**

Despite some improvements to services as a result of health policies and recommendations, more progress is required to ensure that health services make reasonable adjustments to reduce both direct and indirect discrimination of people with intellectual disability.

## Introduction

People with intellectual disability have a higher prevalence of health problems [1] and the median age of death is 25 years younger than the general population [2]. They are more likely to experience inequalities in accessing health care and to die from preventable causes, possibly as a result of institutional discrimination within health services [3], [4], [5], [6].

A number of qualitative (and mixed design) studies have explored the experiences of individuals with intellectual disability, and their carers, in accessing mainstream health services for physical health problems. They have highlighted barriers to accessing health care, including: communication difficulties, resulting from individuals with intellectual disability being excluded from consultations [7], [8], [9], failure of General Practitioners (GPs) to conduct health reviews, review medication and conduct blood tests and investigations [10], lack of health promotion and screening [10], [11], [12] and inadequate knowledge of doctors about the health needs of people with intellectual disability [7], [12], [13], [14], which has contributed to diagnostic overshadowing [9], [14], [15], [16]. Diagnostic overshadowing occurs where signs and symptoms arising from physical or mental health problems are misattributed to the individual's intellectual disability, and can lead to delayed diagnosis and treatment. In hospitals, concerns have been reported about the denial of basic needs such as lack of support during meal times or toileting [16], [17], [18]; problems in the administration of medication [18], and inadequate discharge arrangements [15], [16]. In addition, studies have reported a lack of support offered to carers [19], disregard for information provided by carers [15], and unrealistic expectations of carers to take on care giving responsibilities on the ward [17], [18].

Several studies concluded that patients with intellectual disability received suboptimal care, and were denied appropriate treatment [7], [9], [13]. Health professionals frequently exhibited negative attitudes and behaviour towards individuals with intellectual disability [7], [15], [16], including questioning whether the person was worthy of surgical treatment, due to discriminatory judgements about the person's quality of life [16], [19].

In England, a number of recommendations and initiatives to improve access to health services, for people with intellectual disability, were introduced following an independent inquiry into health care access [5]; (Table 1.) This includes the requirement of health services to make reasonable adjustments to enable individuals with intellectual disability to access services, as stipulated by the Disability Discrimination Act (1995). The Act requires that information about treatment options, complaints procedures and appointments, are provided in an accessible format, and that any processes and procedures that may discriminate people with disability, either directly or indirectly, should be modified so that they are easier to use. Health services are also obligated to take steps to promote equality for people with intellectual disability and to ensure that their needs are addressed even if that involves more favourable treatment. In addition, since 2008, GPs in England have been incentivised to provide annual health checks for people with intellectual disability. Health checks have been shown to increase detection of serious unmet health needs such as cancer, dementia and heart disease. They also increase detection of minor problems such as sensory impairments, which are often treatable and can significantly improve the individual's quality of life [20], [21].

**Table 1:** Key recommendations to improve health care access for people with intellectual disability in England [5].

Recommendations
1.Health services are required to make “reasonable adjustments” in accordance with disability equality legislation, and that effective systems are in place to deliver and monitor whether reasonable adjustments are being made
2. Health services should collect data (e.g. on whether the person has an intellectual disability) to enable health services to identify and track people with intellectual disability through care pathways
3.Commissioning of primary care services to provide annual health checks in 2008
4. Liaison staff to work with primary care to improve the quality of health care for people with intellectual disability across a range of health services
5. Establishment of the Learning Disabilities Public Health Observatory in (established in 2010). Their role is to publish reports on aspects of healthcare for people with intellectual disability such as progress of annual health checks and avoidable premature deaths
6. Undergraduate and postgraduate training for health professionals to include mandatory training in intellectual disability
7. Family and carers should be involved as partners in the provision of treatment and care. They should be provided with information, practical advice and service coordination

In this study, we examine the healthcare experiences of people with intellectual disability and their carers across a range of health care services, and discuss the extent to which implementation of legislative changes and initiatives has improved access to care, the lessons that appear to have been learnt so far, and what further progress is required to make services more equitable. This study was one of two studies, which were part of a PhD examining the experiences of stigma and discrimination reported by people with intellectual disability. The other study was a cross sectional study investigating the relationship between self reported stigma and health outcomes.

### Aims and objectives

The aim of this qualitative study was to examine the extent to which adults with mild or moderate intellectual disability (described in this study as “patients”) and carers believe that their needs are being accommodated by health services. The objectives were to address the following questions:

1. What are patient's and carer's experiences of health services, including both positive and negative experiences, and to what extent do they believe they are receiving unfair treatment or are being discriminated against by health services?
2. What barriers are there to accessing help from health services?
3. How can health services continue to be improved so that they are more attuned and responsive to the needs of people with intellectual disability and their carers?

## Methods

### Ethics statement

Ethical approval was obtained from the West London Research Ethics committee (3) in November 2010, which reviews research conducted on patients within the National Health Service. The committee approved the participant information sheets, consent forms and interview schedules that were used in the study. The research was conducted according to the protocol approved by the ethics committee. The participants (both individuals with intellectual disability and carers) were required to give informed written consent prior to participating in the study. Accessible information sheets and consent forms that used simple text and pictures were given to individuals with intellectual disability to aid comprehension about the study. Capacity to consent to the study was determined by whether the participants met the criteria for the Mental Capacity Act (2005), which applies to England and Wales. This Act requires that participants are able to understand the nature and procedures involved in the study, the advantages and disadvantages of taking part, including adverse events, are able to weigh up the pros and cons of taking part and are able to communicate this decision. Carers assisted in the process of obtaining consent, but did not consent on the behalf of the participants. Participants who were unable to give consent were not included in the study. Refusal to participate in the study did not affect access to treatment or other services.

### Recruitment

Patient-carer dyads, that is, pairs of two closely associated individuals, were recruited. In this study, each dyad consisted of a person with intellectual disability and a carer who knew them well. A focus on dyads allows an understanding of the individual needs of the participants, and the interactions and dynamics that occur between service users and their carers [22]. Dyads have not been previously used to examine people with intellectual disability's experiences of mainstream physical health services, although they have been used in the study of psychiatric services [23]. As part of the recruitment process, community intellectual disability services, day centres and voluntary organisations were approached at eleven sites in the UK (5 in London and 6 outside London (Sussex, Surrey, Somerset, Kent, Nottinghamshire and Lincolnshire)). The recruitment of participants was facilitated through members of staff at the different organisations who approached patients and carers, and through invitation letters or newsletters that were sent by some of the services giving information about the study. Some participants from difficult to reach ethnic minority groups were recruited through snow-balling techniques.

Half the sample was comprised of participants who responded to invitation letters or were approached by staff. The remainder were purposively selected on the basis of cultural and ethnic backgrounds and nature of health problems, in order to obtain a more diverse sample and a wider range of perspectives.

### Inclusion and exclusion criteria

Participants with mild or moderate intellectual disabilities who were aged between18 and 65 were included in the study. The level of intellectual disability was not directly assessed but was based on information from clinical notes and information provided by the referrers. Participants unable to give informed consent were excluded. Both informal carers (e.g. relatives, friends) and paid carers were included. All of the carers had to know the person well (for at least 2 years). In order to be eligible for the study, both the carer and the patient with intellectual disability had to agree to participate in the study.

### Procedures

The study was conducted between May 2011 and September 2012. Semi-structured interviews were conducted with patients and carers separately (by AA), in order to give the patient an opportunity to voice their views and concerns. However, there was some flexibility in the procedures as some patients wanted their carers present at their interview, or their carers needed to be present in order to facilitate the interview due to complex communication needs. All the interviews were held at participants' homes apart from four that were held at a voluntary organisation. The interviews with the patients with intellectual disability lasted between 20 and 45 minutes and the interviews with carers lasted between 30 and 60 minutes.

A structured data collection form was used to collect some basic socio-demographic and clinical data about the participants. Semi-structured interview schedules for patients and carers were used to prompt the researcher of questions or topics to explore. These were initially developed from the literature review but were then modified following input from health and social care professionals and individuals with intellectual disability at two consultation groups that were held at a community intellectual disability service at one of the main participating sites. The topics addressed in the interview schedule included any experiences of health services that were particularly memorable; positive and negative experiences of different types of health services (e.g. primary care, hospitals, dental care, community intellectual disability services); any experiences of unfair or discriminatory treatment; whether complaints were made; the impact of negative experiences on subsequent use of health services; the influence of legislative changes on healthcare experiences; and how health services could be improved so that they meet the needs of carers and patients with intellectual disability.

The interviews were audio-taped and field notes of the interviews were made. Complete data saturation was achieved with no new topics or themes emerging in the final few interviews. The interviews were transcribed verbatim. All the participants were given a £20 gift voucher to thank them for their time.

### The researcher's position

Reflections on the primary researcher's (AA) position and its potential influence on the conduct of the study and interpretation of the results are presented in Box 4.

### Sample characteristics

The total of 29 participants were made up of 14 patient and carer dyads and one single carer (patient declined to participate on the day). Six of the dyads were recruited from two inner London boroughs (Camden and Islington), five dyads (and 1 carer) were recruited from a borough in East London (Newham), one from a borough in South East London (Bromley) and two dyads were recruited from outside London (Somerset and Lincolnshire). Four of the dyads were recruited through snow-balling techniques.

The patients with intellectual disability were between 23 and 57 years of age; seven were male and seven female. Nine were of White British or White Other backgrounds, two were of Asian Indian and three were of Asian Pakistani origin (See Table 2). Ten had a mild intellectual disability and four had a moderate intellectual disability. Three of the patients had Down syndrome, one had cerebral palsy and two had autistic spectrum disorders. The patients had a range of health problems including epilepsy (2), hydrocephalus (2), sensory impairment (4), diabetes (2), hypertension (2), asthma (2) and mental health problems (3).

**Table 2:** Summary of socio-demographic and clinical information for all the dyads.

Dyads	Interview details	Patient Identification Number	Socio-demographic details of patient	Carer identification Number	Socio-demographic details of carer
No.1	Conducted at home. Participants interviewed separately	Patient 1 (P1)	Male, aged 25, White British. Mild ID. Lives in family home	Carer 1 (C1)	Female, aged 72, White British, married. Mother of patient
No.2	Conducted at home. Carer present at interview with patient and facilitated interview	Patient 2 (P2)	Female, aged 26, moderate ID, White British. Lives in family home	Carer 2 (C2)	Female, aged 52, White British, separated. Mother of patient
No.3	Conducted at home. Participants interviewed separately	Patient 3 (P3)	Male, aged 24, White Other (Spanish). Mild ID. Lives at home	Carer 3 (C3)	Female, aged 42, White Other (Spanish), married. Mother of patient
No.4	Conducted at home. Carer present at interview with patient	Patient 4 (P4)	Male, aged 25, White Other (Mixed).Mild ID. Lives in family home	Carer 4 (C4)	Female, aged 52, Irish, divorced. Mother of patient
No.5	Conducted at home. Carer present at interview with patient	Patient 5 (P5)	Female, aged 28, White British. Moderate ID. Lives in family home	Carer 5 (C5)	Female, ages 68, White British, Single. Mother of patient
No.6	Conducted at home. Participants interviewed separately	Patient 6 (P6)	Female, aged 31, Irish. Mild ID. Lives in supported housing	Carer 6 (C6)	Female, aged 60, Irish, married. Mother of patient
No.7	Conducted at home. Carer present at interview with patient	Patient 7 (P7)	Male, aged 30, White British. Mild ID. Lives in supported housing	Carer 7 (C7)	Female, 28, White British, married. Paid carer.
No. 8	Conducted at home. Carer present at interview with patient. Advocate present	Patient 8 (P8)	Male, aged 57, Indian, married. Mild LD. Lives in family home.	Carer 8 (C8)	Female, aged 57, Indian, married. Wife of patient
No.9	Conducted at voluntary organisation. Interviews conducted separately	Patient 9 (P9)	Female, aged 38, White British. Mild ID. Lives in family home.	Carer 9 (C9)	Female, aged 54, White British, divorced. Mother of patient
No.10	Conducted at home. Interviews conducted separately. Advocate present at both interviews	Patient 10 (P10)	Male, aged 42, Indian, married. Mild ID. Lives in family home	Carer 10 (C10)	Female, aged 40, Indian, married. Wife of patient
No. 11	Conducted at home. Carer present at interview with patient. Advocate also present	Patient 11 (P11)	Male, aged 29, Pakistani. Mild ID. Lives in family home	Carer 11 (C11)	Female, aged 53, Pakistani, divorced. Mother of patient
No. 12	Conducted at voluntary organisation. Interviews conducted separately	Patient 12 (P12)	Female, aged 46, White British. Moderate ID. Lives with partner	Carer 12 (C12)	Male, aged 52, White British, partner of patient
No. 13	Conducted at home. Interviews conducted separately. Advocate present at both interviews	Patient 13 (P13)	Female, aged 23, Pakistani. Moderate ID. Lives in family home	Carer 13 (C13)	Female, aged 43, Pakistani, separated. Mother of patient
No.14	Conducted at home. Carer present at interview with patient. Advocate also present	Patient 14 (P14)	Female, aged 29, Pakistani. Mild ID. Lives in family home	Carer 14 (C14)	Female, aged 57, Pakistani, married. Mother of patient
No. 15	Conducted at home with carer only	Did not take part	Patient is 27 years old, had mild ID and lives in family home	Carer 15 (C15)	Female, aged 52, Indian, married, mother of patient

The carers were between 28 and 72 years of age. Most of the carers were mothers of the patients, apart from one who was a paid carer and three who were partners. Only one male carer took part. He was the patient's partner and had borderline intellectual functioning. An advocate who knew the family well, and who was involved in facilitating access to health care, was present at interviews with five dyads. The advocate also assisted with interpreting where the carers or service users had difficulty understanding English.

### Analysis

Analysis of the transcripts was performed using thematic analysis, based on the method described by Braun and Clarke [24]. For this study, an essentialist stance was taken, which reports the participants' experiences as a reflection of reality. Initially the interview transcripts were read several times by the researcher in order to become familiar with the data. This was followed by coding of the data, using the software package NVivo (version 10). NVivo was used to manage the data set but the actual coding was done by the researcher. All transcripts were analysed to derive initial codes, which were applied to segments of the data and closely reflected the raw data (inductive analysis). Following this, all the data extracts relating to the same code were collated together. The third stage involved grouping the different codes into potential themes. The fourth stage involved reviewing the codes, and their grouping into themes with another member of the research team (KS), who also independently coded four transcripts, in order to assess the validity of the coding frame and themes. Following this, some of the codes and themes were re-named and re-organised. Once the final coding frame was identified, the reliability of the coding frame was assessed by another researcher (KS) using two transcripts. The average Cohen's kappa coefficient was 0.82, indicating a good level of agreement between the two raters.

## Results

Eight themes were identified relating to the three objectives and are grouped under: Barriers in health care access; discrimination from health services; and good practice (Table 2). These themes are discussed in detail and illustrated with interview extracts below. The notation used in the brackets refers to the participant identification numbers shown in Table 3 (C denotes carers and P denotes patients).

**Table 3:** Summary of themes and subthemes.

Topic	Theme
**Topic A: Barriers to health care access**	Theme 1. Problems with communication
	Theme 2. Problems with accessing help
	Theme 3. Problems with how health professionals relate to carers
	Theme 4. Complexity of the healthcare system and lack of support for carers
**Topic B: Discrimination from health services**	Theme 5. Substandard care of people with intellectual disability
	Theme 6. Problem with staff attitudes, knowledge and behaviour
**Topic C: Good practice**	Theme 7. Examples of good practice and improvements in services
	Subtheme 8. Suggestions for improvement

### Barriers to health care access

#### I. Problems with communication

Problems with communication were discussed by 12 patients with intellectual disability and 12 carers. Some patients felt ignored by clinicians during consultations or “were talked over” if their carer was present. Staff failed to modify and adapt their communication to the needs of the patient such as asking too many questions, speaking too quickly, giving too much information and not giving the person enough time to respond. Some patients with intellectual disability complained of not understanding what was being said, or not being understood themselves. Several carers reported that the patient's communication difficulties or lack of confidence, affected their ability to express their concerns. Most patients found it helpful to have their carer or an advocate present at the consultation, in order to facilitate communication and understanding:

*“I'd like to know what's happening...I'd like to say something...I think the doctors like talking to the parent about what's happened to the child, but I need to know. I think parents go first and daughter or son goes second about what's happening, I need to know... I don't want to be left behind and I want the doctors to speak to me and my mum together” (P5).*

Patients with intellectual disability and carers reported not being adequately informed about diagnoses, procedures and medication regimes. This included failure of doctors to inform patients of potential side effects of medication, what to do in response to side effects, and lack of information about the dosing and duration of medication. Lack of information or understanding led to patients becoming frightened or feeling pressurised to have treatment.

*P9: “And it was quite uncomfortable, because they put my legs in the stirrup”*

*Interviewer: “Did they explain this to you before the operation?”*

*P9: “No, No”*

*Interviewer: “How did you feel?”*

*P9: “Scary, and they gave me an epidural and I didn't like that because it made my legs go numb and I have problems with my legs.”*

*Interviewer: “Did they explain that they were going to do this before the procedure?”*

*P9: “No, no. They didn't explain nothing really”*

*“He does feel pressurised by them...he's had the operation, it hasn't worked. Now they're saying that they want to do it again. And he never went to the last appointment because he felt they were going to bully him into doing it” (C15; mother)*.

Information was rarely provided in an accessible format that could be understood by patients:

*“No, they just said that I had to sign something... that was it, it was like a consent form. They gave me a little booklet beforehand but it wasn't like an easy read one” (P9).*

#### II. Problems with accessing help

Problems with accessing help were discussed by eight patients with intellectual disability and 12 carers. Carers raised concerns about difficulties in accessing timely support, and of unmet health needs in the patient. Patients with intellectual disability were denied GP home visits if they refused or could not attend the GP surgery; the GP was sometimes perceived to be unhelpful, particularly for social issues. For some carers, getting help from services only occurred during a crisis and was perceived to be a constant battle.

Carers complained of the difficulty in obtaining information about what services were available, and lack of clarity about referral pathways and how services were structured. Obtaining help was compounded by disputes between services about eligibility issues and who should take responsibility for the patient. In the UK, community intellectual disability services are multidisciplinary services that provide expertise in health and social care issues that affect people with intellectual disability. In our study, five carers reported having no knowledge of these services or only being referred recently, suggesting inadequate transition from child to adult services, and their GPs failed to subsequently refer them to specialist services. Of note, in all of the five dyads, the participants were South Asians, which raises the question whether health services are meeting the needs of this group.

*“When he left the hospital at the age of 16, he should have had a good transition to the adult services, but it didn't happen. It's not just to me but I see this happen to lots of people. They're not getting their support plans made, they seem to be slipping through the net” (C15; mother).*

*“I think it's very confusing as to where services are and how it's structured. How you can access services and what is available to you. There's no clear thing that says if you're in this situation, this is what's available to you and this is what you can do...it's like an unknown world out there” (C7; paid carer).*

Several carers who did not speak English as their first language reported that language was a significant barrier to accessing help. They were ignored at consultations, little consideration was given to their views and Information about the patient was frequently not shared with them. The language barrier also prevented some carers from accessing basic support such as assistance completing benefit forms. Many health services failed to provide these carers with an interpreter, which perpetuated their feelings of marginalisation.

*“I have been to many meetings with the doctors but because my English isn't good, I couldn't say what I wanted to say. They never had a translator there at the meetings for me” (C8; wife).*

#### III. Problems with how health professionals relate to carers

Nine carers and one patient with intellectual disability reported problems in the relationship between health professionals and carers. Carers criticised staff for not sharing information or consulting them about clinical decisions. The carer's knowledge of managing the patient's health problems was often disregarded by staff. Carers who were proactive in managing the patient's health care were regarded as “pushy” or over-protective. One paid carer reported feeling like a “piggy in the middle” between hospital staff and the relatives of the service user:

*“We were sort of piggy in the middle kind of thing, going from him, speaking to his mum, and speaking to social services and trying to find out information from the hospital. It was very difficult to find out information from the hospital... And we are asking questions and they are very secretive, um, I understand the confidentiality aspects of it, but somebody needs to know what's happening” (C7; paid carer).*

Carers reported not receiving copies of clinic letters and therefore had to ensure they attended appointments where important decisions were going to be made, which was not always practicable. Some carers felt embarrassed when their presence at appointments was questioned by staff who failed to understand why an adult may need to be accompanied:

*“And then when you go in with your son they always look at you if to say God what sort of mother's like that, going in with a man that size” (C4; mother).*

#### IV. Complexity of the health care system and lack of support for carers

Challenges in negotiating complex health care systems were discussed by 15 carers and nine patients with intellectual disability. Carers thought that it was important to be proactive, as they could not rely on health services taking the initiative in ensure that the patient's needs were met. Consultations were pressured for time. In particular, it was difficult to address concerns within the constraints of the ten minute slot allocated with the GP, which meant that this had to be carefully managed. Some patients with intellectual disability found it difficult to use a telephone based system. Mobility problems or cost of transportation made it difficult for some patients to attend hospital appointments. Carers had learned to manage the health care system over a number of years by acquiring knowledge of how different systems worked. Being articulate and knowledgeable about the patient's health problems was an advantage and usually led to more positive health experiences but carers also reported feeling intimidated because of lack of knowledge and being unable to question clinical decisions.

*“I've had to learn it as a whole technique of how to manage it, what to do about it...So you have to learn to play the game, and that means information, using your own experience”(C5; mother).*

Several carers declared that managing the health care needs of the patient was emotionally draining and resulted in stress, poor emotional wellbeing, and exacerbation of health problems in the carer. Sometimes this led to certain health needs in the patient remaining unmet. Some carers had little support from family or services. Others were able to obtain valuable assistance from voluntary or advocacy groups.

*“I think it's put a ceiling on what I can cope with so, for example, her teeth and her feet and toes. I think that's gone on longer untreated because I just can't cope with it any more. Any more appointments, any more processes, any more people to relate to, any anything” (C5; mother).*

*“It's been very detrimental to my health, the last few years, the way he's been because it's not easy seeing your child suffering from a life threatening condition and not being supported“ (C15; mother).*

Carers reported that they did not have the time or the confidence to make complaints. One carer reported that she had instigated a complaint four years ago but it had not been resolved. Two carers reported that when they complained about poor medical care received by their loved ones, they received a minor acknowledgement that mistakes had occurred but no further action was taken. One carer reported that she had asked a solicitor to investigate further but could not afford the legal costs to pursue the case further. Patients were unlikely to complain because they did not know what the procedures for making a complaint were or, did not think that it would make a difference, or were worried that complaining could have an adverse impact on future care.

### Discrimination from health services

#### I. Substandard care of people with intellectual disability

Twelve patients with intellectual disability and 14 carers gave examples of poor health care provision, including distressing or traumatic experiences. In many of the examples that were given, it is likely that the experiences are not specific to people with intellectual disability and that other patient groups could have had similar experiences, such as the elderly or those with physical disability. Examples included poor continuity of care such as inadequate follow up and being reviewed by a different doctor each time, leading to the prescription of incorrect medication and to unnecessary investigations; lack of adequate discharge arrangements from hospital such as an occupational therapy assessment of the home; and investigations and treatments being delayed or lacking altogether. Sometimes carers had to be persistent in negotiating with the clinicians for investigations to be conducted. In one case, the carer alleged that the patient's behavioural difficulties were misattributed to her intellectual disability, resulting in the doctors refusing to investigate further. This led to a serious medical diagnosis (spinal cord compression) being missed, culminating in permanent irreversible neurological damage.

*“They were ignored all of the time they were in there. It took about eight weeks for a diagnosis and in that time they were trying to get them back home, sort of not looking into anything else, assuming that it was them not being compliant. But actually there was serious underlying problem, in which they didn't do a ...an MRI scan” (C7; paid carer).*

Concerns were also reported about the neglect of basic needs on hospital wards, such as staff not responding to requests of support to use the toilet because they were too busy. Sometimes this had long term consequences for the patient.

*“Too busy to see to you right now, If you pressed the buzzer...it would be a couple of hours until somebody came round...Or if they wanted to go to the toilet...it wasn't for another hour, an hour and a half until somebody came back to do that. The result of that has been reduced continence...they were left to just soil themselves. And now that's become a habit, and now they're back in their own home, it's a thing we've got to work on” (C7; paid carer).*

Half the participants thought that the patient had been discriminated against or treated poorly because of their intellectual disability.

*“My Nan sort of had diabetes as well, but you could see the way they talked to her and the way they talked to me, it was completely different” (P9).*

*“But I do feel, I never thought of it before, but would a man at 23 have had all...he wouldn't have had the same treatment. I think of my brother for instance, if something like that happened to him he wouldn't put up with that” (C 4; mother).*

Some participants acknowledged that patients with intellectual disability were inadvertently treated poorly because staff had misjudged, or had limited awareness of the patient's abilities and needs. Few health services made reasonable adjustments to accommodate the person's needs, such as the provision of additional support when patients were admitted to hospital.

*“I can't remember which hospital it was but they gave him the menus but he didn't know how to complete the menus...no one explained to him... so when his dinner came it was like a slice of toast...they just gave him the menu and left him to it. Two minutes of someone sitting there saying, do you want a hand mate” (C4; mother).*

*“Another time when she stayed in hospital... she had quite an upsetting time...they didn't provide her with a box to put her (insulin) needles in what so ever, so she left them on the table and a nurse pricked herself and she wasn't very nice to her about it and that obviously upset her...She can appear very capable and very normal and they just sort of take that for granted without really knowing her and finding out her needs” (C9; mother).*

In some circumstances, both carers and patients with intellectual disability did not think they were treated differently, and acknowledged that at times, everybody was treated poorly. However, the patient's lack of understanding about their care meant that they were likely to perceive their treatment differently and more negatively compared to someone without the same difficulties.

*“The thing is we've had some terrible things happen...um... but I don't know if you'd say that they've been worse because of his difficulties... anybody would have experienced it, but for him I think it was more traumatic, so to be fair I don't think in most cases we were treated differently but because of his lack of understanding it, it upset him more” (C4; mother).*

Many participants reported reluctance about returning to hospitals or GP surgeries because of the poor treatment that they received. Some patients were able to change their hospital to one which was perceived to be better. Some patients simply refused to attend appointments but others felt that they had no choice but to return to the service.

*“Well you stop using them...you think they weren't helpful last time, what's the point in going and sometimes you have to work on your thinking and say well give them another chance. Like you do with the GP, you have a barrier wall but you still have to go, but for some people the barrier stays up for such a long time and they miss out and that's wrong” (C15; mother).*

#### II. Problems with staff attitudes, knowledge and behaviour

Five patients with intellectual disability and nine carers recalled incidents when health staff had been impolite or unfriendly towards them. Accounts included being spoken to in an abrupt or condescending manner, staff appearing unwelcoming, using insulting language or appearing disinterested.

*“It's like, (they) come into your room for just a second and they talk to you sometimes like you're a five year old” (P7).*

*“It's like you're not really there and sometimes they don't even look at you and acknowledge you properly. It's like everything else is much more important than anything else you have to say… I felt like they sort of look down on you a bit, it was like we know what we're doing, you don't need to know” (C7; paid carer).*

Several carers remarked that they were surprised and astounded at the lack of knowledge that some members of staff had about conditions associated with intellectual disability such as epilepsy:

*“Well it's a seizure, and he stood there, actually solid, like that, and there was a nursing assistant walking past, and I said he's seizing, and she said, no he's not…Their only knowledge of a seizure is the sort when you roll around on the floor, so I thought they're very ignorant about it...I didn't think that nurses wouldn't know what seizures looked like. It just never dawned on me” (C4; mother).*

*“He probably doesn't know or isn't interested about learning difficulties, he's a medical practitioner...I don't know if as a doctor, if he's heard about autism and Asperger's syndrome, perhaps they're difficult, but you kind of think I wonder if they had because they're certainly not helping him out in anyway” (C1; mother).*

### Good practice

#### I. Examples of good practice and improvements in services

Twelve patients with intellectual disability and 13 carers discussed examples of good practice from health services. Higher levels of satisfaction were associated with staff who had gone “beyond the call of duty” to accommodate the needs of patients.

*“She actually went for an overnight stay and she got very distressed because she went there and she had forgotten her injection...she was so distressed about it so I said go to the ward and explain to them, and when they did, they were so nice... And obviously they could see her needs, they took the time to show her around where she would be staying, and they made another appointment, and you know, she was a different person then because she knew they understood” (C9; mother).*

Other examples included good communication skills, friendly and helpful staff and situations where both the patient and carer felt respected. There were also a few examples of the health care system being flexible and accommodating towards the needs of people with intellectual disability, such as offering longer appointments.

*“It was the first time that a doctor had ever spoken directly to her and although they've always been really nice and helpful, he actually just addressed her only and then only looked at me for support, you know, if she was struggling for an answer. And I just thought he was absolutely amazing, he was so respectful to her and that was really good” (C2; mother).*

There were examples of good care being provided, including GP health checks (completed for half the patients participating), with GP surgeries taking the initiative to arrange these, and the provision of health promotion strategies by community services. There were also examples of good transition of care from children to adult services, good continuity of care, examples where help was obtained quickly and services providing support to carers and patients with intellectual disability. A few carers also commented that there were aspects of health care that were improving, although there was still some way to go:

*“I think that's (inpatient care) got better because they give you a care plan and you answer loads of questions and I think that's got better, saying that we had the menu thing so that means no one actually looks at the care plan” (C4; mother).*

When participants were asked whether they thought that health care had improved in recent years, some responded that either their experience had remained unchanged or had become worse. A few thought that legislative changes in the UK such as the Disability Discrimination Act and the Mental Capacity Act were confusing and did little to improve or clarify things.

#### II. Suggestions for improving care

Eight patients with intellectual disability and nine carers provided suggestions for improvement. Several participants commented that health services could be improved if they provided information in an accessible and easy to read format, or if patients were provided with a health passport or a communication book that enabled clinicians and carers to communicate changes in the treatment plan. Several of the carers commented that services needed to make reasonable adjustments to accommodate the needs of people with intellectual disability. This included people with intellectual disability being invited to see a ward prior to a surgical procedure, and being prioritised in some instances, to avoid having to wait too long before appointments. Other carers suggested computer records should highlight that the person has an intellectual disability in order to alert staff.

*“I think yeah, one of the things would be, when you go into a doctor's surgery, as far as I know if a person's diabetic, it comes up, why not have the same sort of thing, this person has got a learning disability... why not flag it up and maybe there's somewhere they can sit, or to think, perhaps it doesn't matter if you let them go in before someone else, if the situation is stressful” (C1; mother).*

Several participants suggested that staff needed to have better knowledge and training in communication skills and conditions that are relevant for people with intellectual disability. Several participants thought it was important that staff had better awareness of individual needs, including more person centred care.

*“I think it's all down to understanding people really, you know because everyone's so individual and their needs are so individual and unless people are aware of their needs. You know it's easy to mark someone with special needs but do they know their special needs, the most important thing is awareness” (C9; mother).*

A few people suggested that this training would be best delivered by involving patients or carers. Suggestions were also made about having access to a hospital liaison or link nurse with expertise in intellectual disability, who could give advice to clinicians, or patients with intellectual disability should be provided with an advocate.

*“Maybe go on courses to learn how to treat people with disabilities properly. Maybe have training sessions with a person with disability actually involved so they know how to treat them...I think it would be good because the way I've been treated, I don't want other people treated the same. I don't think it's right” (P9).*

*“There should be somebody in every hospital, where some adult or a child with a learning disability is admitted, someone who is an expert could go and assess the situation and may be stay with the person if they haven't got someone and be their advocate, and someone who actually knows what autism is like and what dyspraxia's like so they can” (C4; mother).*

### Comparing themes between patients and carers

#### Between group comparisons

The themes that were most reported by patients with intellectual disability were problems with communication and examples of good practice, followed by the substandard care of people with intellectual disability, and problems with the complexities of the health care system (Table 4). For carers, the most prevalent themes were complexities of the health care system and lack of support for carers, and the substandard care of people with intellectual disability. The least reported themes for both patients with intellectual disability and carers were problems with staff attitudes, knowledge and behaviour and problems with how health professionals relate to carers.

**Table 4:** Examples of agreement and disagreement in the accounts given by carers and patients within each dyad.

Dyad number	Number of themes referred to by patient	Number of themes referred to by carer	Number of themes referred to by both carer and patient	Examples of agreement in accounts by carer and patient	Examples of disagreement in accounts by carer and patient
1	5	8	5	Poor communication skills of GP	Accessing help perceived to be easy by patient and difficult by carer; patient satisfied with health check but carer dissatisfied.
2	2	6	2	High levels of satisfaction with health services; staff perceived as friendly and respectful	None
3	7	7	6	None	Patient reported negative attitudes of health professional and staff not modifying communication skills
4	7	7	6	Distressing experiences in hospital; poor knowledge of staff about epilepsy/ID; staff failing to modify communications skills; staff not consulting with carer	None
5	3	8	3	Staff not talking directly to patient; examples of good practice and friendly/helpful staff	None
6	6	4	4	Positive experiences of primary care and community services	None
7	7	8	7	Staff not spending time with patient on ward and not respecting patient; patient and carer not informed/consulted.	Patient dissatisfied with length of hospital admission but carer thought this enabled discharge arrangements to be made
8	5	5	4	None	Patient satisfied with input from primary care but carer dissatisfied (GP refusing home visits, not investigating health complaints)
9	7	7	6	Satisfaction with primary care; less satisfied with hospital care; examples or poor care and good practice.	None
10	6	5	4	None	Patient satisfied with input from primary care but carer dissatisfied (difficulty in arranging home visits, concerns not taken seriously by GP and carer not consulted)
11	6	4	4	Poor experience of inpatient care and Accident and Emergency department.	Some services perceived to be better by carer and advocate but not by patient
12	2	4	1	Positive experience of primary care and community services	None
13	1	7	1	Health professionals failing to talk directly to patient and not involving patient in discussions	None
14	3	5	3	Satisfied with care received from primary care and hospital services	None

#### Comparing the agreement in the themes within individual dyads

The number of themes that were referred to by both the carer and patient in each dyad was compared (Table 4). Reference was made to at least six themes by both the carer and patient in four dyads (dyads 3, 4, 7 and 9). Agreement within the dyads in the themes did not necessarily mean agreement in the accounts given by the patient or the carer. For example, in dyad 1, both the carer and patient commented that the GP's communication skills were inadequate. However, the patient reported that accessing support had been uncomplicated, whereas his carer reported that eligibility issues had made it difficult to access services. Further examples are given in Table 2. Eight dyads showed agreement in accounts, three showed disagreement in accounts and three were mixed (both agreements and disagreements).

## Discussion

### Summary of findings

In this study we investigated the experiences of health care for physical needs from the perspective of patients with intellectual disability and their carers. A number of patients felt that they were discriminated against, or treated differently because of their intellectual disability. Some of these experiences were due to direct discrimination resulting from negative staff attitudes towards patients and carers and failure to treat patients with respect and dignity. Other experiences were due to indirect discrimination arising from lack of staff awareness of patients' needs, and health services failing to accommodate the needs of people with intellectual disability.

Barriers in accessing health services included communication difficulties experienced by patients due to staff failing to speak directly to them or failing to modify their communication skills; problems accessing services due to lack of information about the availability of local services; poor transition of patients from child to adult services; failure of GPs to refer patients to specialist services; and failure to provide interpreters to non-English speakers. Other barriers included lack of support and involvement of carers in health care decisions.

Many of the participants reported examples of good care and improving practice, such as being invited for health checks, suggesting that some of the initiatives to improve health care access have been successful, although further progress was required. A number of suggestions were made about improving care, including the provision of more training for staff in communication and awareness of the needs of patients with intellectual disability; services making reasonable adjustments to support people with intellectual disability such as the provision of accessible information, use of a health passport or communication book; and measures to improve staff attitudes towards people with intellectual disability.

### Areas where further progress is required

Many of the findings from this study are in line with research cited in the introduction, in suggesting that individuals with intellectual disability and their carers continue to experience barriers in accessing health care, in spite of initiatives to improve access. Areas that particularly need addressing are summarised in Table 5 and include:

**Table 5:** Areas where further improvements are required.

Areas requiring improvement	Recommendations
**General issues**	1.Provision of training for clinical and reception staff on communication skills
	2. Specific training of clinicians on intellectual disability, including addressing diagnostic overshadowing and negative attitudes and discrimination. Ideally delivered by service users and carers
	3.Ensure services are culturally sensitive and interpreters are available if required
	4.Services should have appropriate policies and procedures in place to make reasonable adjustments where required (e.g. longer appointment times, accessible information, use of communication passports)
**Primary care services**	1.Increase awareness of annual health checks amongst people with intellectual disability
	2.Improve information about availability of local resources and services, especially to ethnic minority groups
	3.Ensure that service users with intellectual disability are identified (particularly from ethnic minority groups) and are referred to community intellectual disability services, where appropriate
**Community services**	1.Ensure effective transition from child to adult services
	2.Improve clarity about how services are structured and referral pathways
	3.Resolve disputes over eligibility issues quickly
	4.Carer's assessments to be provided more regularly by social services, with provision of feedback
**Hospital/inpatient services**	1.Carers should be consulted and involved in decisions about service user's care
	2.Involvement of liaison nurse where available
	3.Ensure appropriate discharge arrangements are made
	4.Clinic letters and discharge letters to be copied to named carer

#### I. Support for carers

Several carers in our study reported health problems, including depression. One study reported that carers of people with intellectual disability had a 40 per cent higher prevalence of health problems, and were four times as likely to be suffering from depression, compared to the general population [25]. Some of the family carers in our study admitted that this meant less urgent health needs in the patient were ignored and therefore remained unmet. Some carers reported that they had no access to emotional and financial support, and that carer assessments by social services had been delayed or not offered. Social services need to be more proactive in conducting assessments of carers' needs, and in alleviating the burden placed on carers. General Practitioners also need to identify and treat health problems in carers.

#### II. Support for ethnic minorities and non English speakers

This study found that South Asians were particularly likely to experience inequalities in accessing health care. Such families are often deprived, isolated, and experience racism, language barriers and high levels of stress, and are less likely to be knowledgeable about intellectual disability and services [26]. Families from minority ethnic communities may encounter double discrimination as a result of having a member with intellectual disability, and having to endure racial discrimination and culturally inappropriate forms of care [27], [28]. The stigma of having a child with intellectual disability may lead to carers feeling marginalised by their community, and even being blamed for the child's disability by their own families [29].

There are also misconceptions among service providers that South Asian carers are more likely to be supported by members of the extended family [30], which may be a reason why support is not always offered. In fact, studies show that these carers receive little support from their families, and that other types of informal support, such as that provided by support groups, temples or mosques play only a minor supporting role [31], [32], [33]. In addition, health professionals may hold negative or discriminatory attitudes towards this group. South Asians are more likely to receive a delayed diagnosis for medical problems because their concerns are disregarded. Views about consanguineous marriages causing genetic problems, and even intellectual disability, may result in health professionals appearing unsympathetic. This may alienate families and make them reluctant to approach health services for assistance [26] [34], [35]. Health services need to ensure that they provide culturally sensitive forms of care and provide interpreters in order to reduce the inequalities caused by the language barrier.

#### III. Improve referral pathways to specialist services

In our study we found that five patients (third of the sample) had not been referred (or experienced delays in referral) to specialist services for people with intellectual disability, and that carers had little knowledge of such services. More effective transition arrangements between child and adult services are required, and more resources need to be available to carers, including information translated into other languages, about what local services are available.

#### IV. Improve uptake of health checks

About half the participants in this study reported that they had health checks by their GP. This is similar to UK national statistics of 49 per cent of people with intellectual disabilities receiving a health check between 2010 and 2011 [36]. Although more GPs are offering health checks, more needs to be done to increase the awareness and benefits of health checks among people with intellectual disabilities, in order to improve uptake [15].

#### V. Health services need to make more reasonable adjustments

Although there were some examples of services making reasonable adjustments, such as providing a longer appointment slot, and inviting patients to see the ward before surgery, more progress needs to be made by health services to ensure that reasonable adjustments are made in order to reduce both indirect and direct discrimination of people with intellectual disability. Adjustments that could be incorporated by mainstream services include easy read (accessible) clinic letters, and information on medication and procedures; the use of a communication or health passport to communicate health needs and treatment changes; allocation of longer appointment slots or offering the first appointment and making appointment booking systems easier to use.

#### VI. More training needs to be provided to doctors and health care staff on issues relevant to people with intellectual disabilities

There were examples of poor treatment, diagnostic overshadowing and negative staff attitudes towards individuals with intellectual disability, suggesting that more needs to be done in ensuring that health professionals receive adequate training. One positive example of training is the online module in intellectual disability produced by the General Medical Council in the UK, which is aimed at providing doctors with the knowledge and skills required to effectively communicate and treat people with intellectual disabilities [37]. This resource is freely available and could be used more widely as a teaching aid for health professionals across a range of disciplines.

### Strengths and limitations of study

The use of dyads has provided a rich and detailed picture of health experiences from different perspectives, including similarities and differences in perspectives. Although efforts were made to conduct separate interviews with patients with intellectual disability and carers, the carer was present in half of the interviews with patients, which may have influenced the nature of the issues that were discussed. In joint interviews, carers were advised to allow patients to voice their opinions and not to interrupt where possible. Another disadvantage of joint interviews is that personal or sensitive information may be divulged by one participant, which could put the other participant at unease. However, in separate interviews there is also the possibility that confidentiality may be compromised, for example if the patient is informed about discussions that took place with their carer [22], [38]. To prevent the breach of confidentiality, neither the carer nor the patient was given information about the other person's interview.

This study found that in over half the dyads, carers and patients with intellectual disability agreed with each other in the themes and accounts that were given. The comparability of findings between two or more groups may be considered as a form of triangulation, which is an assessment of whether the findings are valid. However, some researchers regard triangulation as an approach to ensuring that data collection and analysis is comprehensive and reflexive, rather than as a test of validity [39]. There were some disagreements in the accounts given by carers and patients. One explanation is that the differences in opinion reflect the different roles and experiences of patients with intellectual disability and carers. The patient's level of cognitive development will also influence the extent to which he or she is able to process and internalise their health care experiences and differentiate between good and inadequate healthcare.

A further strength of the study is the relatively large sample size, as previous qualitative studies investigating health experiences have included fewer participants. We included patients from a range of different backgrounds with both mild or moderate intellectual disability, and varying physical and mental health needs, which provided a diverse sample and a range of different perspectives. There was a relatively large sample of participants from the South Asian community, and the study provides further insight into the experiences of this group. Participants were also recruited from a number of different settings and locations.

One of the limitations of this study is that l almost all of the carers were female and were largely informal carers (parents and partners). The health experiences of male carers and paid carers may be very different. There were no participants from Black (e.g. African or Caribbean) or other Asian backgrounds (e.g. Chinese), and the views of individuals with severe and profound intellectual disability were not considered in this study. The issues raised in this study were also influenced by the interview schedule, which may have limited the exploration of other issues. In addition, the participants who agreed to take part in the interviews may have had more health problems and more negative experiences of health care. Some caution also needs to be given to interpreting that incidents of poor care were due to the patient’s intellectual disability. In the absence of experimental research, we can only conclude that these were perceptions rather than conclusive evidence.

It should also be noted that the primary researcher's (AA) professional and personal background will have shaped the analysis and interpretation of the data (see Table 6).

**Table 6:** Reflections about the conduct of the study.

Stage of research	Reflection
**Pre-research stage**	The primary researcher's (hereafter AA) professional role as a psychiatrist for people with intellectual disability has included acting as a health advocate. She has witnessed patients with intellectual disability receiving poor quality care for physical health problems. This experience, alongside general concerns about inequalities in health care access, influenced the research questions and the study design.
**Data collection**	The use of dyads made it more challenging to recruit participants as both the patient and their carer were required to take part
	Managing interviews where the carer was present at the patient's interview, presented some challenges. Some carers were keen to voice their opinion, and this may have deterred some patients with intellectual disability from volunteering information.
	AA was very mindful of the possibility of a power imbalance between herself and participants, particularly given her professional background. She tried to ensure that her approach was non-judgemental and emphasised that she was in no way responsible for participants' clinical care
	AA's background as a female of South Asian background had some advantages. She was able to recruit people from South Asian backgrounds who may not normally have participated in research. Being female allowed many women to talk freely and openly to her, which they may not have done if the interviewer was male. Conversely, some males (particularly from South Asian communities) were more reluctant to talk to her, possibly because of cultural factors relating to the disapproval of females and males mixing. In addition, AA's prior personal knowledge of some of the issues that affect South Asians may have resulted in less attention being paid to these issues
	In the interviews with patients with intellectual disabilities, frequently closed questions were used due to the difficulty of eliciting responses using open ended questions. Throughout AA had to be conscious of the possibility of suggestibility and acquiescence bias
**Analysis and interpretation**	There were differences in opinion within the research team about the nature of themes identified. The team were able to reach a consensus following discussion
	There is likely to be some subjectivity in the analysis and interpretation of the data resulting from personal experience, biases and assumptions of the researchers

### Implications of the study

Inequity in accessing healthcare for people with disability is a global issue. Recently the World Health Organisation published its “World Report on Disability” [40]. The report makes several recommendations on improving access to health care. Many of these recommendations have already been implemented in the UK in relation to people with intellectual disability, and this study suggests that they have had some impact on improving access to health care for this population. It is important to share this experience with other countries that may be in the process of implementing similar changes, but also to implement these changes more widely so that they are considered for other populations that experience significant barriers to equitable health care, either due to cognitive or communication impairments, or complex health needs. However, one of the lessons learnt so far is that long term commitment is required from both government and health organisations, alongside measures to enforce and evaluate the successful implementation of strategies.

### Directions for future research

Longitudinal qualitative studies where participants are interviewed several times over several months or years may provide more insight into current practice and whether access to health services is improving for patients and their carers. Large scale cross sectional studies on healthcare access would provide more representative data on the prevalence of discrimination and other barriers preventing healthcare access, and could be used to plan local health services.
